# Targeting Microglial HDAC5 Suppresses NLRP3 Inflammation via NF‐κB Deacetylation to Alleviate Ischemic Brain Injury

**DOI:** 10.1002/cns.71161

**Published:** 2026-09-29

**Authors:** Ru‐Yu Liang, Jia‐Qi Zhang, Tian‐Yi Jiang, Li‐Qing Tao, Wen She, Zhou‐Na Sun, Cui Qi, Jun Gao

**Affiliations:** ^1^ Department of Neurobiology, School of Basic Medical Science Nanjing Medical University Nanjing China; ^2^ Center for Novel Target and Therapeutic Intervention (CNTTI), College of Pharmacy Chongqing Medical University Chongqing China; ^3^ Shanghai Key Laboratory of Craniomaxillofacial Development and Diseases, Shanghai Stomatological Hospital & School of Stomatology Fudan University Shanghai China; ^4^ Nantong Fourth People's Hospital Nantong China; ^5^ Department of Cancer Research Center, Nantong Tumor Hospital The Affiliated Tumor Hospital of Nantong University Nantong China

**Keywords:** extracellular vesicles, HDAC5, ischemic stroke, NLRP3, targeted delivery

## Abstract

**Objective:**

Histone deacetylases are implicated in ischemic stroke, yet the role of HDAC5 remains poorly defined. This study aimed to determine the pathological significance of HDAC5 after cerebral ischemia, clarify its involvement in post‐ischemic NF‐κB p65/NLRP3 signaling, and evaluate an HDAC5‐targeted extracellular vesicle (EV)‐based therapeutic strategy.

**Methods:**

HDAC5 expression after ischemic injury was examined in a mouse middle cerebral artery occlusion (MCAO) model by western blotting and immunofluorescence. Its function was assessed by gain‐ and loss‐of‐function experiments in BV2 cells and MCAO mice. Infarct volume, cerebral blood flow, and neurological recovery were evaluated by TTC staining, magnetic resonance imaging (MRI), color Doppler imaging, and behavioral tests. Co‐immunoprecipitation, acetylation analysis, and cytoplasmic/nuclear fractionation were used to explore the underlying mechanism. Human neural stem cell‐derived EVs loaded with miR‐9‐5p and modified with RGD peptide were further tested as a targeted therapeutic approach.

**Results:**

HDAC5 was markedly upregulated after MCAO, localizing mainly to neurons in the ischemic core and microglia in the penumbra, together with increased NLRP3 expression. In BV2 cells, HDAC5 overexpression increased NLRP3 expression, whereas HDAC5 knockdown reduced it. In vivo, AAV‐mediated HDAC5 knockdown decreased NLRP3 levels, reduced infarct volume, improved blood flow recovery, and ameliorated motor deficits after MCAO. Mechanistically, HDAC5 interacted with NF‐κB p65 rather than NLRP3. HDAC5 knockdown increased p65 acetylation and reduced its nuclear translocation, consistent with suppression of NLRP3‐associated inflammatory signaling. Moreover, engineered RGD‐EV:miR‐9‐5p was efficiently taken up by microglia, suppressed HDAC5 and NLRP3 expression, and improved histological and functional outcomes after MCAO.

**Conclusion:**

These findings identify HDAC5 as a previously underappreciated regulator of post‐ischemic inflammatory injury and support a role for the HDAC5/NF‐κB p65/NLRP3 axis in cerebral ischemia. Engineered RGD‐EV:miR‐9‐5p may therefore represent a promising targeted therapeutic strategy for ischemic stroke.

## Graphical abstract

Cerebral ischemia/reperfusion induced HDAC5 upregulation in microglia. Elevated HDAC5 promoted NLRP3‐mediated neuroinflammation via NF‐κB p65 deacetylation. RGD‐EV:miR‐9‐5p delivery suppressed microglial HDAC5 and attenuated ischemic brain injury.

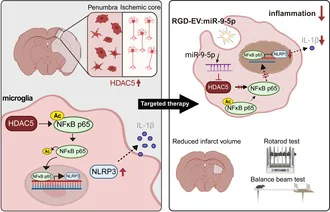

## 1 Introduction

Stroke remains a leading cause of death and long‐term disability worldwide, and ischemic stroke accounts for the majority of cases [1, 2]. Cerebral arterial occlusion interrupts blood supply and deprives brain tissue of oxygen and glucose, triggering excitotoxicity, oxidative stress, inflammation, and ultimately cell death [3, 4]. Although recombinant tissue plasminogen activator (tPA) is currently the only approved pharmacological treatment, its clinical use is restricted by a narrow therapeutic window [5, 6, 7]. Identifying new therapeutic targets and developing effective interventions therefore remain urgent priorities.

Protein acetylation and deacetylation are critical for maintaining central nervous system homeostasis and are profoundly disturbed after ischemic injury [8, 9]. Histone acetyltransferases and histone deacetylases (HDACs) dynamically regulate this balance [10]. Increased deacetylation has been reported after cerebral ischemia, and HDAC inhibitors have shown protective effects in experimental stroke by reducing inflammation, limiting brain injury, and improving neurological recovery [11, 12, 13, 14, 15, 16, 17, 18]. While most previous work has focused on Class I HDACs, increasing evidence suggests that Class II HDACs, which shuttle between the nucleus and cytoplasm, also participate in ischemic pathology [19]. Among Class II HDACs, HDAC5 has attracted increasing attention because of its roles in angiogenesis, axonal regeneration, and inflammatory regulation [20, 21, 22, 23, 24, 25]. Evidence from non‐cerebral disease models further suggests that HDAC5 may be involved in inflammation‐related signaling [23, 26]. However, whether HDAC5 contributes to cerebral ischemic injury, particularly post‐ischemic neuroinflammation, remains unclear. Clarifying this issue may provide new insight into the epigenetic regulation of ischemic brain injury.

In parallel with target identification, effective delivery strategies are also needed for stroke treatment. Extracellular vesicles (EVs) have emerged as promising non‐cellular carriers because of their small size, favorable biocompatibility, and ability to deliver functional RNAs [27, 28, 29, 30, 31]. A pilot clinical study further supported the feasibility of EV‐based therapy in patients with malignant MCAO [32]. To improve targeting efficiency, EVs can be modified with c(RGDyK), which binds integrin αvβ3, a receptor markedly upregulated in vascular endothelial cells and microglia after cerebral ischemia [33].

In the present study, we observed marked upregulation of HDAC5 after ischemia/reperfusion (I/R), together with increased NLRP3 expression. AAV‐mediated knockdown of HDAC5 alleviated ischemic brain injury, supporting HDAC5 as a potential therapeutic target in stroke. Further experiments showed that HDAC5 interacted with NF‐κB p65, and that HDAC5 inhibition increased p65 acetylation and reduced its nuclear translocation, which was associated with decreased NLRP3 expression and inflammasome activation. Based on these findings, we developed an RGD‐modified EV system loaded with miR‐9‐5p and showed that RGD‐EV:miR‐9‐5p protected against post‐ischemic brain injury, at least in part, in association with modulation of the HDAC5/NF‐κB p65/NLRP3 signaling axis.

## 2 Materials and Methods

### 2.1 Mice and Cerebral Ischemia Model

C57BL/6 male mice (8–10 weeks, 25–30 g) were obtained from the Animal Core Facility of Nanjing Medical University (Nanjing, China). Cerebral ischemia was induced using a middle cerebral artery occlusion/reperfusion (MCAO/R) model. Briefly, the right common carotid artery (CCA), internal carotid artery (ICA), and external carotid artery (ECA) were exposed, the ECA was ligated, and a silicone‐coated 6–0 nylon filament was inserted through the ECA stump into the ICA to occlude the middle cerebral artery (MCA). After 1 h of occlusion, the filament was withdrawn to allow reperfusion, and the incision was sutured. Sham‐operated mice underwent the same procedure without MCA occlusion.

For demarcation of the ischemic core and penumbra, a clear boundary between the infarct core and normal cortex was observed 24 h after reperfusion. The peri‐infarct region located 2 mm from this boundary was defined as the ischemic penumbra.

### 2.2 TTC Staining and Laser Doppler Imaging

At 24 h after I/R, mice were euthanized and brains were collected, briefly frozen at −20°C for 11 min, and cut into 2 mm coronal sections. Sections were stained with 1% 2,3,5‐triphenyltetrazolium chloride (TTC) at 37°C for 20 min, fixed in 4% paraformaldehyde (PFA), photographed, and analyzed using ImageJ to calculate infarct volume.

For cerebral blood flow monitoring, the skull was exposed under anesthesia before MCAO, and blood flow was recorded using a Doppler flow monitor. Doppler imaging was used to confirm successful occlusion after filament insertion and to assess blood flow recovery 24 h after reperfusion.

### 2.3 Neurological Function Score

Neurological deficits were assessed 24 h after I/R using an established 18‐point neurological scoring system evaluating motor function, reflexes, sensory responses, and balance [34]. Scoring was performed by an independent blinded investigator.

### 2.4 Cell Culture

ReN cells (Millipore, USA) were cultured in DMEM/F12 medium (Life Technologies, USA) supplemented with 2% B27 (Life Technologies), 10 ng/mL bFGF (Abcam, Canada), and 20 ng/mL EGF (Abcam, UK). HEK293T and HT‐22 cells were cultured in high‐glucose DMEM supplemented with 10% fetal bovine serum (FBS) (PAN, Germany). BV2 cells were maintained in DMEM/F12 containing 10% FBS. All cells were cultured at 37°C in 5% CO_2_.

### 2.5 Transfection of RNA (siRNA or miRNA) and Lentiviral Infection

Small interference RNA (si*Hdac5*) and a miRNA mimic (miR‐9‐5p) were synthesized by GenePharma Co. (Shanghai, China). Cells were transfected using Lipofectamine 2000 reagent (Invitrogen) according to the manufacturer's protocol. Lentivirus‐based small hairpin RNA (shRNA) was obtained from Obiosh Co. (Shanghai, China) and was used to infect BV2 cells according to the manufacturer's protocol. The RNA sequences are shown in Table S1.

### 2.6 Construction of HDAC5 Recombinant Plasmid and Its Transfection

Mouse adrenal tissue was collected for total RNA extraction using Trizol, and *Hdac5* cDNA was amplified by PCR. After confirmation by agarose gel electrophoresis, the PCR product was digested with HindIII and Kpn1 and ligated into the pEGFP‐C1 expression vector. Recombinant plasmids were transformed into DH5α cells and verified by restriction digestion and sequencing.

For transfection, BV2 cells were seeded in 6‐well plates and transfected with 4 μg *Hdac5* plasmid at approximately 80% confluence using Lipofectamine 2000.

### 2.7 EVs Isolation

ReN cells were cultured in 10 cm dishes for 72 h, and conditioned medium was collected for EV isolation by differential ultracentrifugation. Briefly, the medium was sequentially centrifuged at 300 *g* for 10 min, 1200 *g* for 20 min, and 10,000 *g* for 30 min at 4°C to remove cells and debris. The supernatant was then ultracentrifuged at 140,000 *g* for 90 min at 4°C using an L‐80XP ultracentrifuge (Beckman Coulter, USA) with an SW32Ti rotor. The pellet was resuspended in PBS, ultracentrifuged again at 140,000 *g* for 90 min, and finally resuspended in 0.22 μm‐filtered PBS. EV concentration was determined using a BCA Protein Quantification Kit (Pierce, USA).

### 2.8 Conjugation of Ligands to EVs and miR‐9‐5p Incorporation

As previously described [35], 3 nmol dibenzocyclooctynyl‐sulfo‐N‐hydroxysuccinimide ester (DBCO‐sulfo‐NHS; Sigma, USA) was incubated with 500 μg EVs for 4 h at room temperature. Excess reagent was removed using a 100 kDa ultrafiltration tube (Millipore). DBCO‐conjugated EVs were then reacted with azide‐modified c(RGDyK) or control c(RDGyK) peptide (SciLight Biotechnology, China) via copper‐free click chemistry. For imaging, Cy5.5 azide (0.3 nmol; Lumiprobe, USA) was added simultaneously. Reactions were incubated overnight at 4°C on a rotary mixer, followed by washing in PBS and ultracentrifugation at 140,000 *g* for 90 min.

For miRNA loading, cholesterol‐conjugated and 2′‐Ome‐modified miR‐9‐5p mimic or miR‐NC (1 nmol) was incubated with 100 μg EVs (or RGD‐EVs) in PBS for 1 h at 37°C to allow membrane insertion. After washing and ultracentrifugation at 140,000 *g* for 90 min, modified EVs were resuspended and stored at −80°C.

### 2.9 Oxygen Glucose Deprivation/Reoxygenation (OGD/R) Assay

HT‐22 cells were pre‐cultured and incubated with 40 μg EVs for 24 h. Cells were then subjected to oxygen–glucose deprivation in glucose‐free medium in a tri‐gas incubator (1% O_2_, 94% N_2_, 5% CO_2_) for 6 h, followed by reoxygenation in complete medium under normoxic conditions (95% air, 5% CO_2_) for 24 h. Samples were then collected for RT‐PCR analysis of microRNA expression.

### 2.10 Stimulation of BV2 Cells

To assess the effect of EV:miR‐9‐5p, BV2 cells were treated with 20 μg/mL EV:miR‐9‐5p, EVs, or EV:miR‐NC for 24 h, followed by stimulation with 1 μg/mL LPS for another 24 h before western blot analysis.

For NLRP3 inflammasome activation, BV2 cells were pretreated by lentiviral infection or miR‐9‐5p transfection, washed with PBS, and incubated with Opti‐MEM (Gibco) containing 500 ng/mL LPS (Sigma) for 23 h, followed by 10 μM nigericin (MedChemExpress, USA) for 1 h before sample collection.

### 2.11 Adeno‐Associated Virus (AAV) Injections

Mice were anesthetized with isoflurane and mounted on a stereotaxic apparatus. After scalp incision and exposure of the skull, burr holes were drilled at the designated coordinates relative to bregma. Then, 1 μL of AAV (titer: 8.01 × 10^12^) was injected at each of the following coordinates: (+0.5 mm, 3.0 mm, 1.75 mm) and (−1.58 mm, 1.0 mm, 1.25 mm). Control mice received the same volume of control AAV at the same coordinates. Robust viral expression was observed 14–21 days after injection.

### 2.12 EV Administration and NIRF Imaging

For targeting experiments, mice received tail‐vein injection of 100 μg RGD‐EV or RGD‐EV:miR‐9‐5p at 12 h after I/R; control mice received an equal volume of PBS. EVs were labeled with Cy5.5 for imaging. After 24 h, brain, lung, heart, liver, spleen, and kidney were harvested, fixed in 4% PFA, and subjected to near‐infrared fluorescence (NIRF) imaging. For behavioral studies, mice received AAV injection 2 weeks before I/R, followed by tail‐vein injection of 100 μg EVs at 12 h after I/R and once daily for the next 3 days.

### 2.13 Dual‐Luciferase Reporter Assay

Potential target genes of miR‐9‐5p were predicted using TargetScan (Release 8.0). DNA fragments containing either the wild‐type (WT) or mutant (MUT) miR‐9‐5p binding site within the Hdac5 3′‐UTR were synthesized and cloned into the pmirGLO dual‐luciferase reporter vector to generate the Hdac5‐WT and Hdac5‐MUT reporter constructs, respectively. For the luciferase reporter assay, HEK293T cells were seeded into 24‐well plates and co‐transfected with either the Hdac5‐WT or Hdac5‐MUT reporter construct together with a miR‐9‐5p mimic or a negative control mimic (NC mimic). Twenty‐four hours after transfection, cells were lysed, and firefly luciferase and Renilla luciferase activities were sequentially measured using the Dual‐Luciferase Reporter Assay System (Promega) according to the manufacturer's instructions. Relative luciferase activity was calculated as the ratio of firefly luciferase activity to Renilla luciferase activity and was normalized to the corresponding control group.

### 2.14 Protein–Protein Molecular Docking

To explore the potential interaction between HDAC5 and NF‐κB p65, protein–protein docking was conducted using the HDOCK web server (http://hdock.phys.hust.edu.cn/), which integrates template‐based and template‐free docking strategies. NF‐κB p65 was used as the receptor and HDAC5 as the ligand. Global docking was performed without specifying binding sites in advance. Docking models were ranked according to the HDOCK docking score, and the top‐scoring complex was selected for structural analysis. The predicted interaction model was visualized using PyMOL.

### 2.15 Western Blot Analysis

For tissue and cell samples, proteins were extracted in RIPA buffer (Beyotime, China) containing protease inhibitor (Pierce), centrifuged at 12,000 *g* for 15 min at 4°C, and quantified by BCA assay. Equal amounts of protein were mixed with 5× SDS loading buffer (Yeasen, China), boiled at 95°C for 10 min, separated by SDS‐PAGE (Bio‐Rad, USA), and transferred to PVDF membranes (Millipore, USA). Membranes were incubated with primary antibodies overnight, followed by horseradish peroxidase‐conjugated secondary antibodies, and visualized by chemiluminescence (Bio‐Rad). Band density was quantified using ImageJ.

Proteins in supernatants were extracted using the methanol‐chloroform method. For nuclear/cytoplasmic fractionation, extraction was performed according to the manufacturer's instructions (Beyotime). The following antibodies were used: anti‐HDAC5 (Santa Cruz, USA), anti‐IL‐1β, anti‐NLRP3, anti‐cleaved caspase‐1, anti‐acetylated‐lysine, anti‐IgG, anti‐NFκB p65, anti‐H3 (CST, USA), anti‐TSG101, anti‐Alix (Abcam, UK), and anti‐β‐Actin (Bioworld, China). Detailed information on the antibodies is provided in Table S3.

### 2.16 Immunoprecipitation (IP)

Cells were lysed in IP lysis buffer supplemented with protease inhibitors and kept on ice for 20 min. After centrifugation, the supernatants were collected for immunoprecipitation. Equal amounts of protein were incubated overnight at 4°C with anti‐HDAC5 antibody (Santa Cruz), anti‐acetylated‐lysine antibody (Cell Signaling Technology), anti‐NF‐κB p65 antibody (Santa Cruz), or the corresponding species‐matched IgG as a negative control. The mixtures were then incubated with Protein A/G beads (Santa Cruz) for 4 h at room temperature. After three washes with lysis buffer, the bound proteins were eluted in SDS loading buffer by heating at 95°C for 10 min and subjected to western blotting. For detection of p65 Lys310 acetylation, acetylated proteins were immunoprecipitated with an anti‐acetylated‐lysine antibody and then immunoblotted with an acetyl‐NF‐κB p65 (Lys310) antibody.

The primary antibodies used for western blotting of immunoprecipitated proteins included anti‐HDAC5 (Santa Cruz), anti‐NLRP3, anti‐NF‐κB p65, anti‐β‐Actin, and acetyl‐NF‐κB p65 (Lys310) antibody (Affinity, USA). Because NF‐κB p65 migrates at a molecular weight close to that of the IgG heavy chain, a universal secondary antibody recognizing both heavy and light chains was used to minimize interference from IgG bands (Abmart, M21008; 1:5000).

### 2.17 Immunofluorescence Staining and Confocal Imaging

For tissue immunofluorescence, mice were perfused with PBS followed by 4% PFA, and brains were collected and cryosectioned into 40 μm slices. Sections were permeabilized with 0.3% Triton X‐100 for 30 min, blocked with 3% BSA for 2 h, and incubated with primary antibodies overnight at 4°C. After washing with PBST, sections were incubated with Alexa 488‐, Alexa 594‐, or Alexa 647‐conjugated secondary antibodies (Invitrogen) for 1 h at room temperature, counterstained with Hoechst 33342, and mounted with ProLong Antifade Reagent (Invitrogen). Images were captured using an FV‐1200 confocal microscope (Olympus, Japan).

For cell staining, cells were grown on poly‐D‐lysine‐coated glass coverslips and imaged using a 60× oil objective. Imaging parameters were kept constant, and relative fluorescence intensity was quantified.

Antibodies used were anti‐NeuN (Millipore), anti‐HDAC5 (Santa Cruz), anti‐γH2A.X (CST), anti‐Iba‐1 (M) (Abcam), anti‐Iba‐1 (R) (CST), anti‐GFAP (Invitrogen, USA), anti‐NLRP3 (Abcam), and anti‐ASC (Proteintech).

### 2.18 Behavioral Test

Behavioral tests were performed on Days 3, 5, and 7 after MCAO/R, including the rotarod and balance beam tests. For the rotarod test, mice were trained for 3 days before I/R. During testing, the rod accelerated from 0 to 40 rpm over 260 s, with a total duration of 300 s. Latency to fall was recorded, and the mean of three trials was used. For the balance beam test, mice were trained for 3 days before I/R to cross a 1 cm diameter, 1 m long beam. The time required to traverse the beam was recorded, and the mean of three trials was used. Mice unable to complete the task were assigned a maximum time of 60 s.

### 2.19 Magnetic Resonance Imaging (MRI)

MRI scans were conducted using a 7.0 T animal MRI scanner. Following behavioral studies, mice from each group were randomly chosen for MRI scanning (T2‐weighted imaging). The main scanning parameters were as follows: T2‐weighted imaging, repetition time (TR)/echo time (TE) of 2500.0 ms/35.0 ms, a field of view (FOV) of 2.20 × 2.00 cm, a matrix of 256 × 256, and a slice thickness (SI) of 0.8 mm. ImageJ software was utilized to calculate and analyze the post‐I/R infarct volume. The ischemic lesion volume was determined as the hemispheric lesion volume (HLV) after adjusting for the hemispheric occupancy effect [36]. HLV% = [contralateral total hemispheric volume − (total lesion hemispheric volume − total ischemic lesion volume)]/contralateral total hemispheric volume × 100%.

### 2.20 Statistical Analysis

Data are presented as mean ± SEM. We used GraphPad Prism software (GraphPad Software, USA) to perform statistical analyses. Two‐group comparisons were conducted using a Student's *t*‐test, and comparisons across multiple groups were conducted using one‐way analysis of variance (ANOVA) followed by a Tukey test. For data involving two independent variables, including viral treatment and surgical condition, two‐way ANOVA was used followed by appropriate post hoc multiple comparisons. A *p*‐value < 0.05 was considered statistically significant.

## 3 Results

### 3.1 HDAC5 Is Upregulated in a Cell Type–Specific Manner After I/R, Accompanied by Increased NLRP3 Expression

Brain sections from mice following I/R were stained using TTC to measure the infarcted area on the right side (Figure 1A). In addition, laser doppler monitoring of blood flow showed a blockage in the right hemisphere following I/R (Figure 1B). Neurological scoring further confirmed functional deficits at 24 h post‐I/R (Figure S1A). Given that hippocampal neurons, particularly those in the CA1 region, are highly sensitive to ischemia and hypoxia [37], NeuN and γ‐H2A.X immunofluorescence staining demonstrated significant neuronal loss accompanied by DNA double‐strand breaks in this region (Figure S1B). Following I/R, the ischemic core and surrounding penumbra regions were isolated (Figure S1C) for protein analysis. Western blot results showed that HDAC5 expression increased at 48 h, which persisted up to 72 h (Figure 1C,D). These findings were further confirmed by immunofluorescence staining (Figure S1D,E).

**FIGURE 1:**
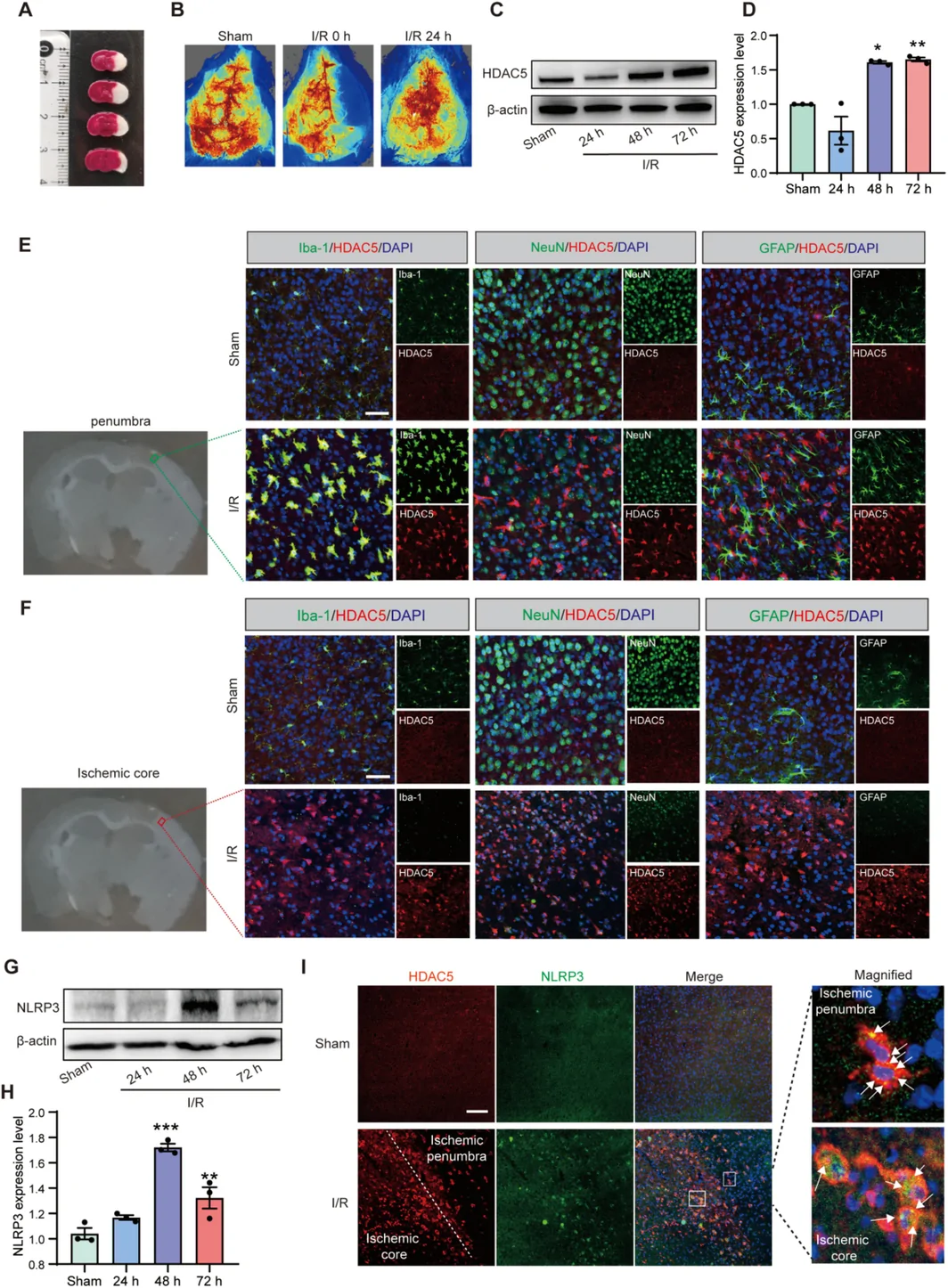
Altered expression levels and distribution of HDAC5 and NLRP3 after I/R. (A) Representative brain image of TTC staining 24 h after I/R. (B) Representative 2D Laser Scatter Images of the mouse brain. (C) Western blot analysis of HDAC5 at different time points after I/R. (D) HDAC5 quantification, normalized to β‐Actin and presented as a relative change compared to sham group (*n* = 3). (E) Co‐labeled fluorescence images of microglia (Iba‐1), neuron (NeuN) and astrocyte (GFAP) with HDAC5 in the ischemic penumbra region of the cerebral cortex. (F) Co‐labeled fluorescence images of microglia (Iba‐1), neuron (NeuN) and astrocyte (GFAP) with HDAC5 in the ischemic core region of the cerebral cortex. All experiments were performed 48 h post‐I/R. The same cerebral cortex location was observed in the sham‐operated control group, and blue color indicates nuclei. Scale bar 50 μm. (G) Western blot analysis of NLRP3 at different time points after I/R. (H) Quantification of NLRP3 was normalized to β‐Actin and presented as a relative change compared with the sham group (*n* = 3). (I) Fluorescence staining analysis of brain sections from the ischemic core region and the junction of the ischemic penumbra; HDAC5 (red), NLRP3 (green), and Hoechst nucleus (blue). White arrows indicate the colocalization of HDAC5 and NLRP3. Scale bar 100 μm. All data are expressed as mean ± SEM. *p < 0.05; **p < 0.01; ***p < 0.001 by one‐way ANOVA.

Cell‐type‐specific analysis revealed distinct localization patterns of HDAC5. In the ischemic penumbra, HDAC5 was predominantly upregulated in Iba‐1–positive microglia (Figure 1E), whereas in the infarct core, HDAC5 was mainly localized in NeuN‐positive neurons (Figure 1F). No significant co‐localization with GFAP‐positive astrocytes was observed in either region (Figure 1E,F), indicating cell‐type‐specific regulation of HDAC5 after I/R.

Given that post‐ischemic injury is closely associated with inflammatory responses, we next examined NLRP3 expression. Western blot and immunofluorescence analyses demonstrated that NLRP3 protein levels were significantly elevated after I/R, peaking at 48 h (Figure 1G,H; Figure S1F), coinciding with HDAC5 upregulation. Notably, NLRP3 exhibited a punctate distribution pattern in cells following ischemia (Figure 1I; Figure S1G), suggesting enhanced activation status. Together, these results indicate that HDAC5 upregulation temporally correlates with increased NLRP3 expression following I/R.

### 3.2 HDAC5 Promotes NLRP3 Expression and Contributes to Brain Injury After I/R

To determine whether HDAC5 regulates NLRP3 expression, we first overexpressed (OE) *Hdac5* in BV2 cells. *Hdac5* OE significantly increased NLRP3 protein levels (Figure 2A–C). Conversely, *Hdac5* knockdown (KD) using siRNA or lentiviral shRNA markedly reduced NLRP3 expression (Figure S2A–D). BV2 cells were treated with LPS and nigericin, which resulted in a rapid increase in NLRP3 expression. Consistent with the above results, *Hdac5* KD led to a decrease in the expression of NLRP3 (Figure 2D,E). Immunofluorescence staining further showed that *Hdac5* KD markedly reduced the formation of punctate NLRP3 aggregates (Figure 2F), indicating suppression of NLRP3 activation.

**FIGURE 2:**
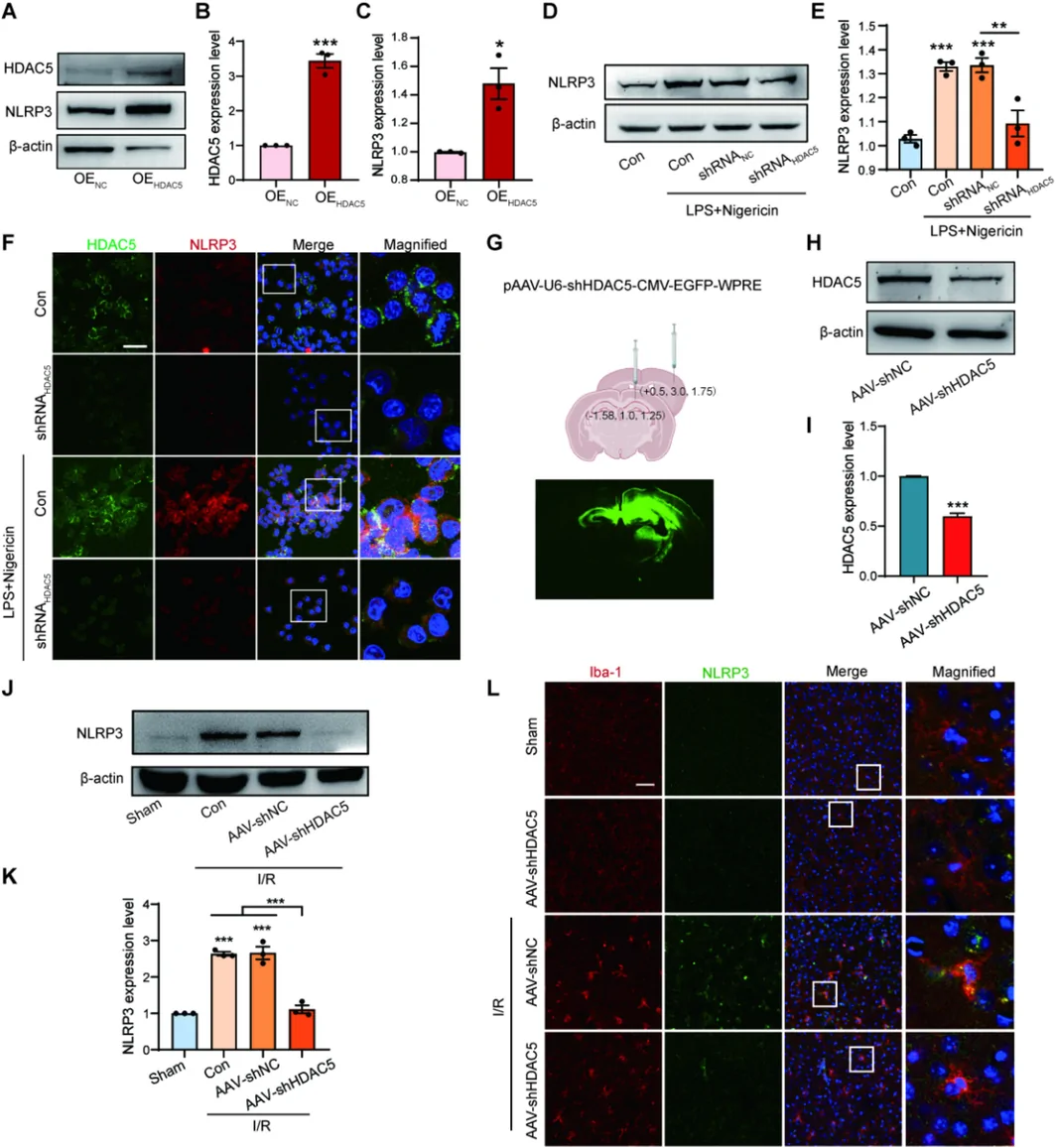
HDAC5 Is involved in regulating the expression of NLRP3 protein and activation of NLRP3 inflammasome. (A) WB analysis of HDAC5 and NLRP3 after *Hdac5* OE. (B, C) Quantification analysis of HDAC5 (B) and NLRP3 (C), (*n* = 3). (D) Western blot analysis of NLRP3 following *Hdac5* KD in response to LPS and nigericin stimulation. (E) Quantification of NLRP3 protein, normalized to β‐Actin and presented as a relative change compared to the sham group (*n* = 3). (F) Immunofluorescence staining of HDAC5 (green) and NLRP3 (red) in BV2 cells after treatments: Blue color indicates nuclei. Scale bar 50 μm. (G) Stereotactic injection of AAV‐shHDAC5 (or AAV‐shNC) was performed with the following coordinates: 0.5 mm anterior to bregma, 3.0 mm right, and 1.75 mm depth from the cranial surface; and 1.58 mm posterior to bregma, 1.0 mm right, and 1.25 mm depth from the cranial surface. Fluorescence imaging of brain sections at the two injection sites, with green indicating areas of viral infection. (H, I) Western blot detection (H) and quantification (I) of AAV‐shHDAC5 KD efficiency, (*n* = 4). (J) Protein immunoblotting analysis of NLRP3 after ischemic modeling in mice injected with AAV‐shHDAC5. (K) Quantitative analysis of NLRP3 protein form (g), (*n* = 3). (L) Fluorescence images of microglia (Iba‐1, red) in the ischemic penumbra area co‐labeled with NLRP3 (green), scale bar 50 μm. All data are expressed as mean ± SEM. *p < 0.05; **p < 0.01; ***p < 0.001. Student's *t*‐test analyzed results shown in (B, C, I), (E, K) were analyzed by one‐way ANOVA.

To validate these findings in vivo, AAV‐shHDAC5 was stereotactically injected into the right cortex of mice. Efficient knockdown of *Hdac5* was confirmed by fluorescence imaging and Western blot analysis (Figure 2G–I). Laser Doppler measurements showed that *Hdac5* KD improved cerebral blood flow after I/R (Figure S3A). In addition, γ‐H2A.X staining revealed reduced DNA damage in hippocampal CA1 neurons (Figure S3B,C). Importantly, NLRP3 expression was significantly decreased in the ischemic cortex of AAV‐shHDAC5–treated mice compared with controls (Figure 2J,K). Immunofluorescence further confirmed reduced NLRP3 levels in microglia within the penumbra region (Figure 2L; Figure S2E). These results demonstrate that HDAC5 positively regulates NLRP3 expression and contributes to ischemic brain injury.

### 3.3 *Hdac5* KD Reduces Infarct Volume and Promotes Neurological Recovery After Cerebral Ischemia

To investigate the effects of *Hdac5* KD on motor function in mice subjected to cerebral ischemia, we performed rotarod and balance beam tests (Figure 3A). Baseline assessments conducted 1 day before MCAO surgery showed that AAV injection did not affect motor performance in mice, as evidenced by comparable results in the rotarod and balance beam tests among groups (Figure S3D,E). After MCAO, compared with ischemic mice injected with AAV‐shNC, mice treated with AAV‐shHDAC5 exhibited significantly improved motor function, as indicated by increased time on the rotarod and decreased time required to cross the balance beam on Days 3, 5, and 7 (Figure 3B,C). Following the behavioral experiments, four mice from each group were randomly selected for brain MRI. MRI clearly revealed the infarct areas, and *Hdac5* KD via AAV‐shHDAC5 significantly reduced infarct volume after cerebral ischemia (Figure 3D,E).

**FIGURE 3:**
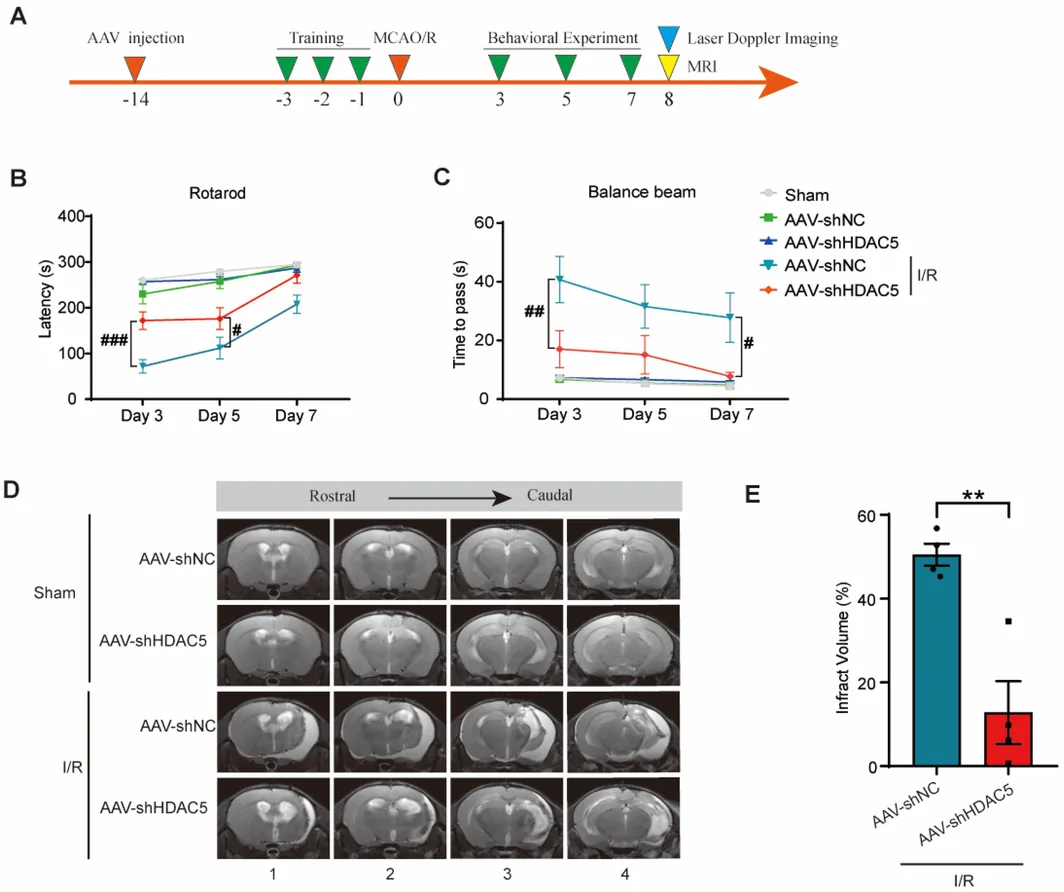
*Hdac5* KD accelerates motor function recovery in I/R mice. (A) Timeline of behavioral experiments, (*n* = 8). (B) On Days 3, 5, and 7, mice in various treatment groups underwent the rotarod test. The residence time of the mice on the rotating sticks is presented. (C) On Days 3, 5, and 7, mice in various treatment groups underwent the balance beam test. The time of the mouse passing through the balance beam is presented. All data are expressed as mean ± SEM. ^#^ *p* < 0.05; ^##^ *p* < 0.01; ^###^ *p* < 0.001 by two‐way ANOVA. (D) MRI imaging of mice. (E) Quantification of infarct volume by MRI (*n* = 4). Data presented as mean ± SEM; **p < 0.01 by Student's *t*‐test.

### 3.4 HDAC5 Promotes NLRP3 Transcription by Regulating NFκB p65 Deacetylation

To elucidate the mechanism by which HDAC5 regulates NLRP3, we first examined whether HDAC5 directly interacts with NLRP3. Immunoprecipitation assays showed no direct interaction between HDAC5 and NLRP3 (Figure 4A), suggesting that HDAC5 regulates NLRP3 through an indirect mechanism. Previous studies have shown that HDAC5 can deacetylate non‐histone proteins, including NF‐κB p65 and STAT3 [26, 38]. To explore whether HDAC5 may interact with NF‐κB p65, molecular docking analysis was performed using the HDOCK server, which predicted a favorable binding model between HDAC5 and NF‐κB p65 (Figure 4B). Consistent with this prediction, co‐immunoprecipitation assays confirmed the interaction between HDAC5 and NF‐κB p65 (Figure 4C; Figure S4A). Furthermore, *Hdac5* knockdown significantly increased NF‐κB p65 acetylation in BV2 cells (Figure 4D,E), as further confirmed by reverse co‐immunoprecipitation (Figure S4B). Because Lys310 is a well‐established acetylation site of NF‐κB p65 and has been widely implicated in regulating its transcriptional activity, we further examined acetylated NF‐κB p65 (Lys310). However, no specific band was detected in any group (Figure S4C), indicating that the increased acetylation of NF‐κB p65 induced by *Hdac5* knockdown is unlikely to be mediated through the Lys310 site.

**FIGURE 4:**
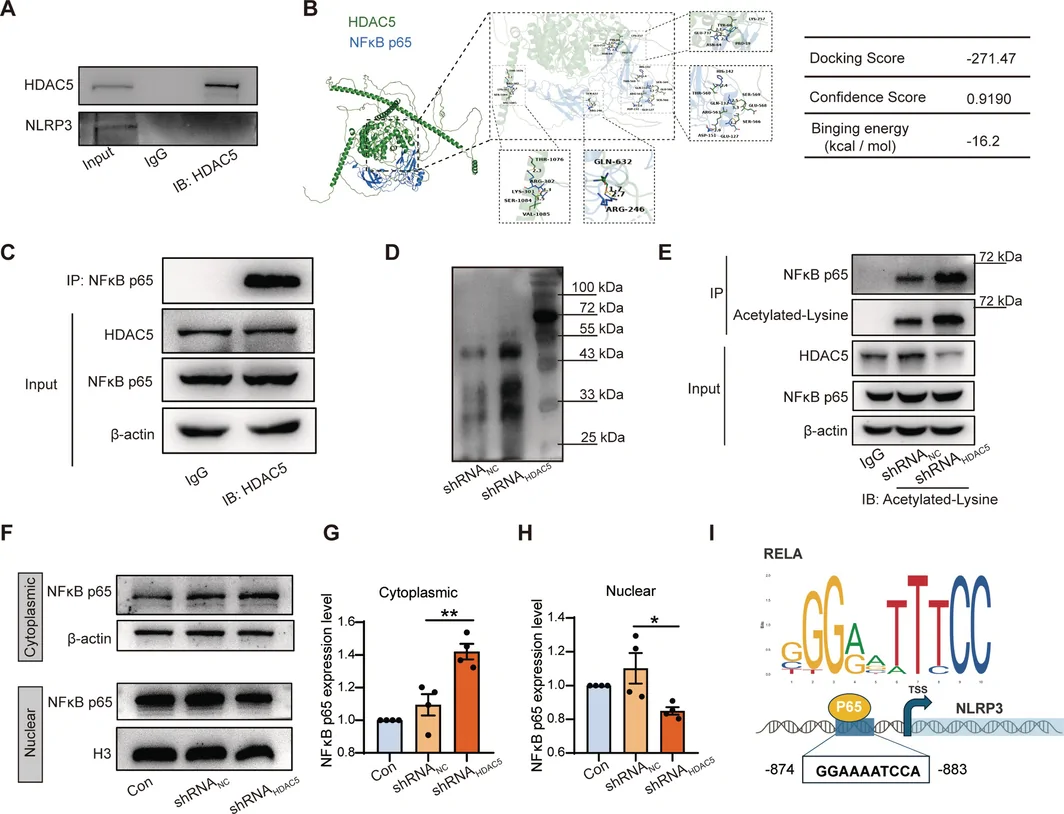
HDAC5 deacetylates NFκB p65 and indirectly regulates NLRP3 expression. (A) BV2 cells were transfected with lentivirus for 48 h and then harvested for Co‐IP assay. (B) Molecular docking analysis predicting the interaction between HDAC5 and NF‐κB p65. The enlarged inset shows the predicted potential binding site. (C) Co‐IP assay showing the interaction between HDAC5 and NF‐κB p65 in BV2 cells. (D) Western blot assay to detect the expression of acetylated proteins after *Hdac5* KD. (E) *Hdac5* KD in BV2 cells and subsequent Co‐IP assay. (F) Western blot assay to detect NFκB p65 protein expression in cytoplasm and nucleus after *Hdac5* KD. (G, H) Quantitative analysis of NFκB p65 protein form (D) (*n* = 4). (I) Schematic of the predicted NFκB p65 (Rela) binding site in the promoter of NLRP3. All quantitative data are expressed as mean ± SEM. *p < 0.05; **p < 0.01; ***p < 0.001 by one‐way ANOVA.

Since activation of NF‐κB p65 requires its nuclear translocation, we next examined its subcellular distribution. *Hdac5* knockdown increased cytoplasmic NF‐κB p65 while reducing its nuclear accumulation (Figure 4F–H), indicating impaired nuclear translocation. In addition, bioinformatic analysis using JASPAR predicted putative NF‐κB p65‐binding sites within the NLRP3 promoter region (Figure 4I). Together, these findings suggest that HDAC5 promotes NLRP3 transcription by interacting with and deacetylating NF‐κB p65., and facilitating its nuclear translocation, thereby functioning as an upstream regulator of NLRP3 through the NF‐κB p65 signaling pathway.

### 3.5 Engineering and Characterization of RGD‐EV:miR‐9‐5p

In addition to identifying novel therapeutic targets, developing effective delivery strategies is critical for ischemic stroke treatment. Extracellular vesicles (EVs) have emerged as promising carriers and have shown protective effects in experimental stroke models. In this study, EVs were isolated from the human fetal neural progenitor cell line ReN, and administration of ReN‐derived EVs significantly alleviated ischemic injury in MCAO mice (Figure 5A–D). RNA‐seq analysis of ReN‐derived EVs revealed enrichment of multiple microRNAs [39]. Based on our previous profiling data of ReN‐derived EV miRNAs, TargetScan‐based prediction, and literature evidence related to cerebral ischemia, we prioritized 10 candidate miRNAs for further validation (Table S2). Among these, particular attention was given to candidates potentially involved in the HDAC5‐related mechanism explored in this study. To highlight the specificity of ReN‐derived EV cargo, EVs from HEK293T cells were used as controls, and cel‐miR‐39 was applied as an external reference for normalization. RT‐PCR analysis showed that miR‐9‐5p was highly enriched in ReN‐derived EVs (Figure 5E). To assess microRNA responsiveness under ischemic conditions, HT22 cells were subjected to oxygen–glucose deprivation (OGD) following EV treatment. Among the candidates, miR‐455‐3p, miR‐455‐5p, and miR‐9‐5p were markedly responsive to OGD, and their expression levels were restored by EV treatment, with miR‐9‐5p showing the most prominent effect (Figure 5F). Bioinformatic prediction suggested a putative binding site between miR‐9‐5p and *Hdac5* (Figure 5G). To further confirm whether *Hdac5* is a direct target of miR‐9‐5p, we performed a dual‐luciferase reporter assay using reporter constructs containing either the wild‐type (WT) or mutant (MUT) *Hdac5* 3′UTR sequence. The results showed that miR‐9‐5p mimic significantly decreased the luciferase activity of the WT reporter, whereas this effect was abolished when the predicted binding site was mutated (Figure 5H), confirming that miR‐9‐5p directly binds to the *Hdac5* 3′UTR. Consistently, miR‐9‐5p overexpression significantly reduced HDAC5 expression in BV2 cells (Figure 5I,J). Together with its enrichment in ReN‐derived EVs and its marked responsiveness under OGD conditions, these findings supported the regulatory effect of miR‐9‐5p on HDAC5 and led us to prioritize miR‐9‐5p for subsequent studies.

**FIGURE 5:**
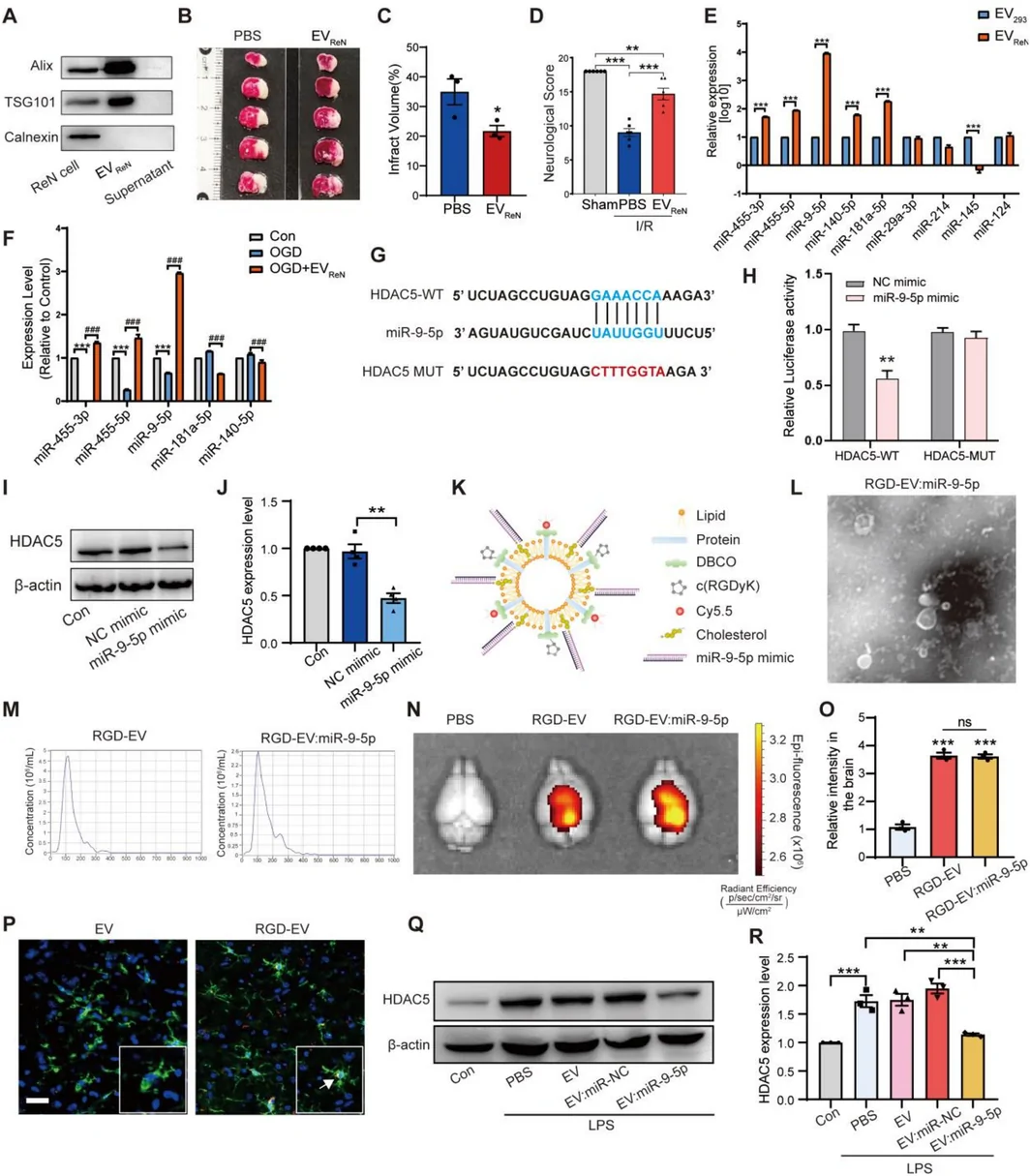
Preparation and characterization of RGD‐EV:MiR‐9‐5p. (A) Western blot was used to detect the expression of the positive markers Alix and TSG101 in EVs. (B) Representative images of TTC‐stained brains from I/R model mice at 24 h after tail vein injection of PBS or EVs. (C) Quantification of infarct volume after TTC staining, *n* = 3. (D) Neurological deficit scores in the sham group, and I/R model mice treated with EVs or PBS. (E) RT‐PCR analysis of the levels of 10 different miRNAs in HEK293T‐derived EVs and ReN‐derived EVs (*n* = 3). (F) After EV_ReN_ was added to HT‐22 cells for 24 h, OGD experiments were performed and 5 miRNAs were detected (*n* = 3). *denotes comparison with the Control group, ***p < 0.001; ^#^denotes comparison between the OGD group and the OGD + EV_ReN_ group, ^###^ *p* < 0.001. (G) The binding sites of miR‐9‐5p on *Hdac5* were predicted by TargetScan. (H) Dual‐luciferase reporter gene assay was used to verify the binding between miR‐9‐5p and *Hdac5*. (I) BV2 cells were transfected with miR‐9‐5p mimics, and after 48 h, the expression of HDAC5 in the cells was analyzed by Western blot. (J) Quantification of HDAC5 (*n* = 4). (K) Schematic of RGD‐EV, labeled with Cy5.5, and loaded with cholesterol‐modified miR‐9‐5p. (L, M) TEM imaging (L) and NTA (M) of RGD‐EV: MiR‐9‐5p; scale bar 200 nm. (N, O) MCAO/R mice were injected with Cy5.5‐labeled RGD‐EVs or RGD‐EV: MiR‐9‐5p, and Cy5.5 levels in the brain were analyzed 24 h later. Representative brain NIR fluorescence images (N) and quantification of intracerebral fluorescence intensity (O) are shown, *n* = 3. (P) After ischemia modeling in mice, tdTomato‐labeled EVs were injected into the tail vein and perfused 24 h later. Fluorescent staining was performed to analyze whether EVs had entered into microglia in the ischemic region; red indicates EVs, green indicates microglia, and the scale bar is 25 μm. (Q, R) BV2 cells were pretreated with PBS, EV (or EV: MiR‐NC, or EV: MiR‐9‐5p) for 24 h before LPS treatment. The expression of HDAC5 (Q) in the cells were determined by Western blot analysis, and quantification of HDAC5 (R), *n* = 3. Data are expressed as mean ± SEM, *p < 0.05; **p < 0.01; ***p < 0.001 by one‐way ANOVA.

To construct a targeted delivery system, miR‐9‐5p was chemically modified with cholesterol at its 3′ end to enable anchoring onto EV membranes via hydrophobic interactions. RGD targeting peptide (or Cy5.5 fluorescent label) and miR‐9‐5p were subsequently conjugated to EVs using a two‐step method, generating RGD‐EV:miR‐9‐5p (Figure 5K). Transmission electron microscopy (TEM) and nanoparticle tracking analysis (NTA) confirmed that EV morphology and size were not altered after modification (Figure 5L,M). In vivo fluorescence imaging demonstrated that RGD‐EV:miR‐9‐5p efficiently targeted ischemic brain regions (Figure 5N,O), and RGD‐EV can effectively uptake by microglia (Figure 5P). Moreover, treatment with EV:miR‐9‐5p significantly reduced HDAC5 expression in BV2 cells (Figure 5Q,R).

### 3.6 RGD‐EV:miR‐9‐5p Suppresses NLRP3‐Associated Inflammation and Exerts Neuroprotective Effects, Potentially Through Targeting *Hdac5*

Based on the findings in Figure 5 showing that miR‐9‐5p directly targets *Hdac5* and that EV:miR‐9‐5p reduces HDAC5 expression, we next investigated whether EV:miR‐9‐5p modulates the inflammatory response post‐ischemia in association with HDAC5 and its downstream effector NLRP3. To explore this, BV2 cells were pretreated with EV:miR‐9‐5p prior to LPS stimulation. This treatment significantly reduced NLRP3 protein levels under inflammatory conditions (Figure 6A,B), suggesting that miR‐9‐5p negatively regulates NLRP3 expression. In vivo, immunofluorescence analysis showed that tail vein injection of RGD‐EV:miR‐9‐5p after I/R significantly attenuated microglial activation and punctate NLRP3 aggregation in the peri‐infarct region compared with PBS‐treated mice (Figure 6C–E). Furthermore, RGD‐EV:miR‐9‐5p treatment significantly decreased infarct volume (Figure 6F), improved neurological scores (Figure 6G), and reduced lesion size as confirmed by MRI (Figure 6H,I). These findings indicate that RGD‐EV:miR‐9‐5p confers neuroprotection possibly by suppressing the HDAC5/NLRP3 signaling axis.

**FIGURE 6:**
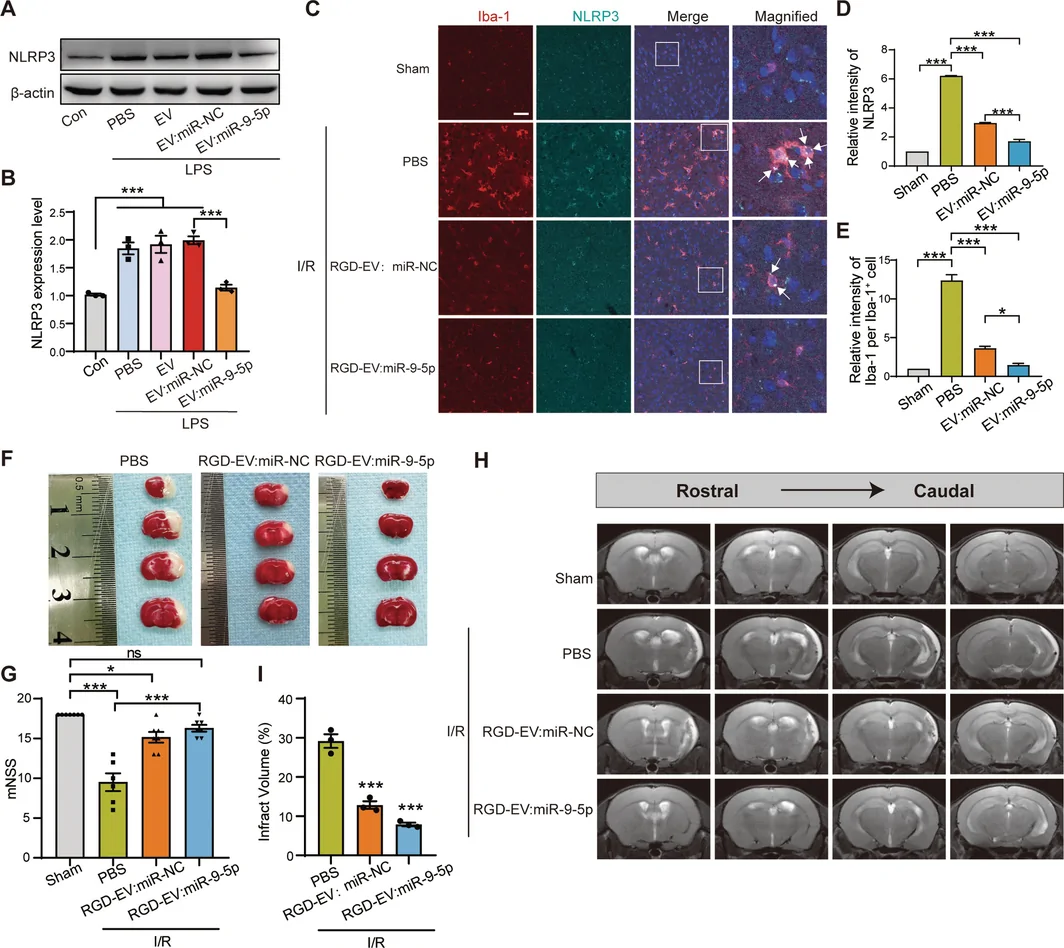
RGD‐EV:miR‐9‐5p inhibits NLRP3 inflammatory activation in I/R mice and rescue post‐ischemic brain injury. (A) BV2 cells were pretreated with PBS, EV (or EV: miR‐NC, or EV: miR‐9‐5p) for 24 h before LPS treatment. The expression of NLRP3 in the cells were determined by Western blot analysis. (B) Quantification of NLRP3 from blots in (A), (*n* = 3). (C) Fluorescence analysis of brain slices from MCAO‐modeled mice in the sham group and treatment groups after Iba‐1 (red) and NLRP3 (cyan) staining; scale bar 50 μm. (D) Quantitative analysis of Iba‐1 fluorescence intensity in figure (C). (E) Quantitative analysis of NLRP3 fluorescence intensity (*n* = 4). (F) Representative TTC staining after PBS or different EV administration. (G) Neurological functional scoring of I/R mice after receiving PBS or RGD‐EV:miR‐NC (or RGD‐EV:miR‐9‐5p) administration. (H) MRI of mice after receiving PBS, RGD‐EV:miR‐NC (or RGD‐EV:miR‐9‐5p) administration. (I) Quantification of infarct volume by MRI (*n* = 3). All quantitative data are expressed as mean ± SEM. *p < 0.05, **p < 0.01, ***p < 0.001 by one‐way ANOVA.

## 4 Discussion

HDAC5 is highly expressed in the heart, brain, lung, and skeletal muscle [40], suggesting a potential role in responses to oxygen availability and metabolic stress. Consistently, HDAC5 is rapidly upregulated in tumor cells under hypoxic conditions [41], further supporting its involvement in hypoxia‐related stress responses. During ischemic stroke, brain tissue is exposed to severe hypoxia and glucose deprivation, making HDAC5 a plausible regulator of post‐ischemic injury. However, previous studies examining HDAC5 after cerebral ischemia/reperfusion (I/R) are limited and have produced inconsistent results [42, 43, 44]. In addition, the cell type‐specific distribution and functional significance of HDAC5 after I/R have remained largely unclear. In the present study, HDAC5 expression showed a dynamic temporal pattern, with an initial decrease at 24 h after reperfusion followed by a marked increase at 48–72 h. Moreover, HDAC5 displayed distinct spatial and cellular distributions at 48 h after reperfusion: in the infarct core, HDAC5 upregulation was mainly observed in neurons, whereas in the peri‐infarct penumbra it was predominantly increased in microglia. These findings suggest that HDAC5 may play different roles in different cell populations after stroke, potentially contributing to neuronal injury in the infarct core and inflammatory responses in microglia within the salvageable penumbra.

The NLRP3 inflammasome is a key mediator of sterile inflammation following tissue injury [45, 46]. Previous studies have shown that NLRP3 expression is markedly increased after cerebral ischemia and that its inhibition confers neuroprotection [47, 48], consistent with our observations. Post‐translational modifications, including phosphorylation, ubiquitination, and acetylation, are important regulators of NLRP3‐related inflammatory signaling [49]. While most previous studies have focused on phosphorylation and ubiquitination, the role of acetylation remains less well defined. In the present study, we found that HDAC5 does not directly deacetylate NLRP3. Instead, HDAC5 appears to regulate NLRP3 expression indirectly by interacting with NF‐κB p65, reducing its acetylation level and promoting its nuclear accumulation. Previous studies have demonstrated that acetylation of NF‐κB p65 weakens its DNA‐binding activity and facilitates IκBα‐dependent nuclear export, thereby limiting NF‐κB transcriptional activity [50, 51]. In this context, HDAC5‐mediated deacetylation of p65 would be expected to enhance its nuclear retention and transcriptional activation of downstream inflammatory genes, including NLRP3 [52]. Thus, our findings support the existence of an HDAC5/NF‐κB p65/NLRP3 signaling axis that links epigenetic regulation to post‐ischemic neuroinflammation.

Based on these mechanistic findings, we further explored the therapeutic potential of targeting HDAC5. We developed an EV‐based strategy to deliver miR‐9‐5p to the ischemic brain. Rather than selecting miR‐9‐5p solely based on previous reports, our choice was guided by experimental observations. qPCR analysis revealed that miR‐9‐5p was highly enriched in ReN cell‐derived EVs. Notably, miR‐9‐5p levels were markedly reduced in BV2 cells following OGD stimulation, indicating that it is responsive to ischemia‐like stress. Importantly, treatment with ReN‐EV effectively restored miR‐9‐5p levels under these conditions. Based on this evidence, we further enhanced miR‐9‐5p expression to maximize its therapeutic potential. In line with this strategy, our data showed that miR‐9‐5p overexpression reduced the levels of cleaved caspase‐1 and IL‐1β in BV2 cells under inflammatory stimulation (Figure S5A–C), suggesting a suppressive effect on NLRP3‐related inflammatory activity. To improve targeting efficiency, EVs were modified with RGD peptide, which binds integrin αvβ3, a receptor markedly upregulated in microglia after cerebral ischemia [33]. This design enabled intravenous delivery of miR‐9‐5p to the ischemic region and provided a practical approach for modulating HDAC5‐associated inflammatory signaling in microglia. In the present study, the ischemic brain‐targeting capability of RGD‐EVs was quantitatively demonstrated by in vivo fluorescence imaging (Figure 5N,O). Moreover, the biodistribution and biosafety of the same RGD‐engineered EV platform have been established in our previous studies, which showed enhanced accumulation in the ischemic brain, the expected distribution in major peripheral organs after intravenous administration, and favorable in vivo safety after repeated dosing [33, 39].

Several limitations should be acknowledged. First, although our findings strongly support the involvement of microglial HDAC5 in post‐ischemic inflammation, this study used global AAV‐mediated *Hdac5* knockdown rather than cell type‐specific genetic manipulation. Therefore, the relative contributions of HDAC5 in microglia and neurons cannot be fully distinguished in vivo. Nevertheless, the marked colocalization of HDAC5 with Iba‐1 in the ischemic penumbra, together with the consistent regulation of NLRP3 signaling by HDAC5 in BV2 cells and the microglia‐targeting design of RGD‐EVs, supports a microglia‐associated role of HDAC5 in this process. Future studies using cell type‐specific models will be needed to define this more precisely. Second, although our data support regulation of NF‐κB p65 acetylation by HDAC5, p65‐based rescue experiments were not performed, and the precise acetylation site(s) involved remain to be identified. Therefore, the present study does not definitively establish p65 as the essential mediator of HDAC5‐dependent NLRP3‐related inflammatory signaling, and the possibility that HDAC5 influences p65 function through additional cofactors or indirect mechanisms cannot be excluded. Third, miR‐9‐5p may regulate targets other than HDAC5, and these additional targets may also contribute to the protective effects observed in vivo. Fourth, the long‐term efficacy, biodistribution, and safety of repeated RGD‐EV administration require further evaluation before clinical translation.

In summary, our study identifies HDAC5 as a previously underappreciated contributor to ischemic brain injury and suggests that HDAC5 is involved in post‐ischemic neuroinflammation, at least in part, in association with the NF‐κB p65/NLRP3 pathway. We further demonstrate that intravenously administered RGD‐modified EVs can effectively deliver miR‐9‐5p to the ischemic brain and exert therapeutic effects. These findings provide mechanistic evidence linking HDAC5 modulation to inflammatory regulation after stroke and support the potential of an EV‐based strategy for targeted intervention.

## 5 Conclusion

In conclusion, we demonstrated that HDAC5 levels were significantly elevated after I/R, with increased expression observed in microglia at 48 h. Our data support an association between HDAC5 upregulation and NLRP3‐related inflammatory signaling, potentially through interaction with and deacetylation of NF‐κB p65. As a potential therapeutic strategy for cerebral ischemia, RGD‐EV:miR‐9‐5p modulated HDAC5 expression and alleviated ischemic brain injury, with the in vivo and BV2‐cell findings supporting a role for microglia‐associated inflammatory regulation.

## Author Contributions

Ru‐Yu Liang: writing – review and editing, writing – original draft, visualization, validation, investigation, formal analysis, data curation, conceptualization. Jia‐Qi Zhang: writing – review and editing, writing – original draft, visualization, validation, investigation, formal analysis, data curation. Tian‐Yi Jiang: investigation, formal analysis, data curation. Li‐Qing Tao: writing – review and editing, investigation, formal analysis, data curation. Wen She: investigation. Zhou‐Na Sun: investigation. Cui Qi: investigation. Jun Gao: writing – review and editing, supervision, resources, funding acquisition, conceptualization.

## Funding

This study was funded by the National Natural Science Foundation of China grant (NO. 32371053, 81973308 and 81673416) and the National Key Research and Development Program of China (NO. 2022YFC2705200) to J.G.

## Ethics Statement

All animal experiments followed institutional guidelines and received approval from the Animal Care and Use Committee of Nanjing Medical University (No. IACUC‐2209007). No human samples were used in this study.

## Conflicts of Interest

The authors declare no conflicts of interest.

## Supporting information


**Figure S1:** (A) Neurological deficit scores in I/R modeling mice. (B) Fluorescence analysis of brain sections from sham and I/R mice after staining for NeuN (green), γ‐H2A.X (red), and Hoechst nuclei (blue). Scale bar 50 μm. (C) White ischemic core area tissue and ischemic penumbra tissue 24 h post‐I/R. (D) Fluorescence analysis of HDAC5 (red) and Hoechst nuclei (blue) at different time points. Scale bar 50 μm. (E) Fluorescence intensity of HDAC5 per cell corresponding to G (*n* = 3). The sham groups were used as negative control. (F) Fluorescence analysis of brain sections staining for NLRP3 (green) and Hoechst nuclei (blue); scale bar, 100 μm. (G) Brain sections co‐stained with HDAC5 (red) and NLRP3 (green) in the ischemic core region and ischemic penumbra; blue indicates nuclei. Scale bar 50 μm. All data are expressed as mean ± SEM. *p < 0.05; **p < 0.01; ***p < 0.001. Student's *t*‐test analyzed results shown in (A), (E) was analyzed by one‐way ANOVA.
**Figure S2:** (A) BV2 cells were transfected with 4 siRNAs (*HDAC5* KD), and Western blotting was performed to analyze the protein of HDAC5. (B) Quantification of HDAC5 was normalized to β‐Actin and presented as a relative change compared with the group of control (*n* = 5). (C, D) After *HDAC5* KD in BV2 cells, NLRP3 was analyzed by Western blotting (C) and quantified (D). (E) Quantitative analysis of NLRP3 fluorescence intensity (*n* = 3). Data are expressed as mean ± SEM. *p < 0.05; **p < 0.01; ***p < 0.001 by one‐way ANOVA.
**Figure S3:** (A) Representative 2D laser scattering images of mouse brains after AAV injection. (B) Fluorescence analysis of brain sections from ischemia model mice injected with AAV‐sh*HDAC5* (or AAV‐shNC) stained with γ‐H2A.X (red), green (GFP) represents virus infected cells, blue is nuclear; scale bar 100 μm. (C) Fluorescence quantification of γ‐H2A.X (*n* = 4). (D) Baseline rotarod performance before MCAO surgery (*n* = 5). (E) Baseline balance beam performance before MCAO surgery (*n* = 5). Data are expressed as mean ± SEM. *p < 0.05; **p < 0.01; ***p < 0.001 by Student's *t‐*test.
**Figure S4:** (A) Co‐immunoprecipitation assay validating the interaction between HDAC5 and NF‐κB p65 in BV2 cells. Cell lysates were immunoprecipitated with anti‐HDAC5 antibody or species‐matched control IgG, followed by immunoblotting for NF‐κB p65. IgG heavy chain (~50 kDa) and light chain (~25 kDa) are visible. (B) Reverse co‐immunoprecipitation further confirmed that HDAC5 knockdown increased the acetylation level of NF‐κB p65 in BV2 cells. (C) Co‐immunoprecipitation assay using a pan‐acetylation antibody for immunoprecipitation, followed by immunoblotting with an acetyl‐NF‐κB p65 (Lys310) antibody.
**Figure S5:** (a) BV2 cells were transfected with miR‐9‐5p mimics for 43 h. After 1 μg/mL LPS treatment for 4 h and 10 μM nigericin treatment for 1 h, cells and supernatant were collected. The expression levels of caspase1 p20 and IL‐1β were determined by western blotting analysis. (b, c) Quantification of caspase1 p22 (b), and IL‐1β (c), *n* = 3, Data are expressed as mean ± SEM. *p < 0.05; **p < 0.01; ***p < 0.001 by one‐way ANOVA.
**Table S1:** Small interfering RNA and microRNA information sheet.
**Table S2:** Basis for selecting the 10 candidate miRNAs for validation.
**Table S3:** Antibodies used in this study.

## Data Availability

The datasets generated during and/or analyzed during the current study are not publicly available, but are available from the corresponding author on reasonable request.
