# Supplementary material for: Targeting Microglial HDAC5 Suppresses NLRP3 Inflammation via NF‐κB Deacetylation to Alleviate Ischemic Brain Injury
Source: CNS Neurosci Ther. 2026 Sep 29;32(10):e71161. doi: 10.1002/cns.71161 (PMC13624526; doi:10.1002/cns.71161)
Supplement: Supplementary file 1 [file CNS-32-e71161-s001.docx]

Supplementary Materials for

**Targeting microglial HDAC5 suppresses NLRP3 inflammation via NF-κB deacetylation to alleviate ischemic brain injury**

Ru-Yu Liang, Jia-Qi Zhang, Tian-Yi Jiang, Li-Qing Tao, Wen She, Zhou-Na Sun, Cui Qi, Jun Gao^*^

***Corresponding author:**

Jun Gao, PhD

Stomatological Institute of Integrated Innovation, Fudan University

Shanghai Stomatological Hospital & School of Stomatology, Fudan University

Email: [gaojun_kq@fudan.edu.cn](mailto:gaojun_kq@fudan.edu.cn)

**Fig.S1** (A) Neurological deficit scores in I/R modeling mice. (B) Fluorescence analysis of brain sections from sham and I/R mice after staining for NeuN (green), γ-H2A.X (red), and Hoechst nuclei (blue). Scale bar 50 μm. (C) White ischemic core area tissue and ischemic penumbra tissue 24 h post-I/R. (D) Fluorescence analysis of HDAC5 (red) and Hoechst nuclei (blue) at different time points. Scale bar 50 μm. (E) Fluorescence intensity of HDAC5 per cell corresponding to G (n=3). The sham groups were used as negative control. (F) Fluorescence analysis of brain sections staining for NLRP3 (green) and Hoechst nuclei (blue); scale bar, 100 μm. (G) Brain sections co-stained with HDAC5 (red) and NLRP3 (green) in the ischemic core region and ischemic penumbra; blue indicates nuclei. Scale bar 50 μm. All data are expressed as mean ± SEM. **p* < 0.05; ***p* < 0.01; ****p* < 0.001. Student's *t*-test analyzed results shown in (A), (E) was analyzed by one-way ANOVA.

**Fig.S2** (A) BV2 cells were transfected with 4 siRNAs (*HDAC5* KD), and Western blotting was performed to analyze the protein of HDAC5. (B) Quantification of HDAC5 was normalized to β-Actin and presented as a relative change compared with the group of control (n=5). (C, D) After *HDAC5* KD in BV2 cells, NLRP3 was analyzed by Western blotting (C) and quantified (D). (E) Quantitative analysis of NLRP3 fluorescence intensity (n=3). Data are expressed as mean ± SEM. **p* < 0.05; ***p* < 0.01; ****p* < 0.001 by one-way ANOVA.

**Fig.S3** (A) Representative 2D laser scattering images of mouse brains after AAV injection. (B) Fluorescence analysis of brain sections from ischemia model mice injected with AAV-sh*HDAC5* (or AAV-shNC) stained with γ-H2A.X (red), green (GFP) represents virus infected cells, blue is nuclear; scale bar 100 μm. (C) Fluorescence quantification of γ-H2A.X (n=4). (D) Baseline rotarod performance before MCAO surgery (n=5). (E) Baseline balance beam performance before MCAO surgery (n=5). Data are expressed as mean ± SEM. **p* < 0.05; ***p* < 0.01; ****p* < 0.001 by Student's *t-*test.

**Fig.S4** (A) Co-immunoprecipitation assay validating the interaction between HDAC5 and NF-κB p65 in BV2 cells. Cell lysates were immunoprecipitated with anti-HDAC5 antibody or species-matched control IgG, followed by immunoblotting for NF-κB p65. IgG heavy chain (~50 kDa) and light chain (~25 kDa) are visible. (B) Reverse co-immunoprecipitation further confirmed that HDAC5 knockdown increased the acetylation level of NF-κB p65 in BV2 cells. (C) Co-immunoprecipitation assay using a pan-acetylation antibody for immunoprecipitation, followed by immunoblotting with an acetyl-NF-κB p65 (Lys310) antibody.

**Fig.S5** (a) BV2 cells were transfected with miR-9-5p mimics for 43 h. After 1 μg/mL LPS treatment for 4 h and 10 μM nigericin treatment for 1 h, cells and supernatant were collected. The expression levels of caspase1 p20 and IL-1β were determined by western blotting analysis. (b, c) Quantification of caspase1 p22 (b), and IL-1β (c), n=3, Data are expressed as mean ± SEM. **p* < 0.05; ***p* < 0.01; ****p* < 0.001 by one-way ANOVA.

**Supplementary Table.S1 Small interfering RNA and microRNA information sheet**

| Name | sequence information（5'to 3'） |
| --- | --- |
| HDAC5-2169（siRNA1） sense | GUCCAGUGCUGGUUACAAATT |
| HDAC5-2169（siRNA1） antisense | UUUGUAACCAGCACUGGACTT |
| HDAC5-1164（siRNA2） sense | GGAUGGCACUGUUAUUAGUTT |
| HDAC5-1164（siRNA2） antisense | ACUAAUAACAGUGCCAUCCTT |
| HDAC5-2395（siRNA3） sense | CAGUGUAUGUGCGGAAACATT |
| HDAC5-2395（siRNA3） antisense | UGUUUCCGCACAUACACUGTT |
| HDAC5-3196（siRNA4） sense | CUGGGUUUGAUGCUGUUGATT |
| HDAC5-3196（siRNA4） antisense | UCAACAGCAUCAAACCCAGTT |
| miR-9-5p mimic sense | UCUUUGGUUAUCUAGCUGUAUGA |
| miR-9-5p mimic antisense | AUACAGCUAGAUAACCAAAGAUU |

**Supplementary Table S2. Basis for selecting the 10 candidate miRNAs for validation**

| miRNA | Previous enrichment in ReN-EVs* | Predicted class II HDAC target(s) (TargetScan) | Reported relevance to cerebral ischemia | Basis for selection |
| --- | --- | --- | --- | --- |
| miR-455-3p | No | HDAC4, HDAC7 | No | Predicted to target class II HDACs |
| miR-455-5p | Yes | - | Yes | Enriched in ReN-EVs and reported in cerebral ischemia |
| miR-9-5p | Yes | HDAC4, HDAC5 | No | Enriched in ReN-EVs and predicted to target HDAC4/5 |
| miR-140-5p | No | HDAC4, HDAC5, HDAC7 | No | Predicted to target multiple class II HDACs |
| miR-181a-5p | Yes | HDAC4, HDAC5 | Yes | Met all three criteria |
| miR-29a-3p | Yes | HDAC4, HDAC5 | No | Enriched in ReN-EVs and predicted to target HDAC4/5 |
| miR-214-3p | Yes | - | Yes | Enriched in ReN-EVs and reported in cerebral ischemia |
| miR-145a-5p | No | HDAC4 | Yes | Predicted to target HDAC4 and reported in cerebral ischemia |
| miR-34a-5p | No | HDAC5 | No | Predicted to target HDAC5 |
| miR-124-3p | No | HDAC4, HDAC5 | Yes | Predicted to target HDAC4/5 and reported in cerebral ischemia |

* “Previous enrichment in ReN-EVs” refers to our previous profiling study of ReN-derived EV miRNAs: *Theranostics* 2021;11(13):6507-6521, Fig. 6A.

**Supplementary Table S3. Antibodies used in this study**

| Antibody | Company | Catalog number |
| --- | --- | --- |
| anti-γH2A.X | CST | 9718 |
| anti-HDAC5 | Santa Cruz | 133225 |
| anti-β-actin | Bioworld | AP0060 |
| anti-H3 | CST | 2650 |
| anti-NeuN （M） | Millipore | MAB377 |
| anti-NeuN （R） | Millipore | ABN78 |
| anti-Iba-1 （M） | Abcam | 283319 |
| anti-Iba-1 （R） | CST | 17198 |
| anti-GFAP 647 | Invitrogen | 51-9792-82 |
| anti-NLRP3 （WB） | CST | 15101 |
| anti-NLRP3 （IF） | Abcam | 270449 |
| anti-NLRP3 （Flow） | Abcam | 263899 |
| anti-ASC/TMS1 | Proteintech | 10500-1-AP |
| anti-Caspase1 | CST | 89332 |
| anti-IL-1β | CST | 12242 |
| anti-NFκB p65 | CST | 6956 |
| anti-NFκB p65 | Santa Cruz | 8008 |
| anti-Acetylated-Lysine | CST | 9441 |
| anti-Acetyl-NF-kappaB p65 (Lys310) | Affinity | 1017 |
| anti-IgG | CST | 7076P2 |
| anti-Alix | Abcam | 186429 |
| anti-TSG101 | Abcam | 125011 |
| anti-Calnexin | Abcam | 22595 |
| Goat anti Mouse IgG-HRP | Biolegend | 12478 |
| Goat anti Rabbit IgG-HRP | Biolegend | 13278 |
| Universal type anti-heavy chain + light chain secondary antibody | Abmart | M21008 |
| Alexa Fluor 488（Donkey anti-Rabbit） | Invitrogen | A21206 |
| Alexa Fluor 594（Donkey anti-Mouse） | Invitrogen | A21203 |
